# A sinemydid turtle from the Jehol Biota provides insights into the basal divergence of crown turtles

**DOI:** 10.1038/srep16299

**Published:** 2015-11-10

**Authors:** Chang-Fu Zhou, Márton Rabi

**Affiliations:** 1 Paleontological Institute, Shenyang Normal University, Shenyang 110034, Liaoning, China; 2 Institut für Geowissenschaften, University of Tübingen, Hölderlinstraße 12, 72074 Tübingen, Germany; 3 Department of Paleontology & MTA – ELTE Lendület Dinosaur Research Group, Eötvös Loránd University, Budapest, Hungary

## Abstract

Morphological phylogenies stand in a major conflict with molecular hypotheses regarding the phylogeny of Cryptodira, the most diverse and widely distributed clade of extant turtles. However, molecular hypotheses are often considered a better estimate of phylogeny given that it is more consistent with the stratigraphic and geographic distribution of extinct taxa. That morphology fails to reproduce the molecular topology partly originates from problematic character polarization due to yet another contradiction around the composition of the cryptodiran stem lineage. Extinct sinemydids are one of these problematic clades: they have been either placed among stem-cryptodires, stem-chelonioid sea turtles, or even stem-turtles. A new sinemydid from the Early Cretaceous Jehol Biota (Yixian Formation, Barremian-Early Aptian) of China, *Xiaochelys ningchengensis* gen. et sp. nov., allows for a reassessment of the phylogenetic position of Sinemydidae. Our analysis indicates that sinemydids mostly share symplesiomorphies with sea turtles and their purported placement outside the crown-group of turtles is an artefact of previous datasets. The best current phylogenetic estimate is therefore that sinemydids are part of the stem lineage of Cryptodira together with an array of other Jurassic to Cretaceous taxa. Our study further emphasises the importance of using molecular scaffolds in global turtle analyses.

Cryptodires represent the largest group of living turtles (Testudines) and exhibit a wide range of habitat preferences from terrestrial to freshwater to marine^1^. Their origin and early evolution can be traced back to a diverse assemblage of fossil turtles from the Middle to Late Jurassic to the Early Cretaceous of Asia^2^,^3^,^4^,^5^,^6^,^7^,^8^,^9^,^10^,^11^,^12^,^13^. The sinemydid turtles of the Jehol Biota of Northeast China are of particular interest to cryptodire origins due to their excellent preservation. Over the course of the last two decades, a large number of sinemydid fossils were recovered from the Jehol Biota but very few of them were described. Until now, only four taxa have been reported from western Liaoning and adjacent areas, in particular *Manchurochelys manchoukuoensis, Ordosemys liaoxiensis, Liaochelys jianchangensis* and the soft-shelled turtle *Perochelys lamadongensis*^13^,^14^,^15^,^16^,^17^,^18^,^19^. A fifth taxon, the sinemydid *Xiaochelys ningchengensis* gen. et sp. nov. is described here based on a skeleton from the poorly known Jehol Biota of the Ningcheng Basin, Inner Mongolia.

The Jehol Biota sinemydids are of particular importance for unravelling the sequence of morphological evolution around the base of crown Cryptodira. Sinemydids are traditionally considered to represent part of the stem-lineage of Cryptodira^2^,^3^,^4^,^5^,^6^,^7^,^8^,^9^,^10^,^11^,^12^,^13^ and are therefore critical for reconstructing the ancestral cryptodire morphology.

However, recent morphological analyses failed to reproduce the traditional position of sinemydids on the turtle tree and have tentatively placed them outside of the crown-group of turtles, basal to the pleurodire-cryptodire dichotomy^12^,^13^,^20^. Further hypotheses proposed that sinemydids are stem-chelonioid sea turtles^21^,^22^,^23^,^24^,^25^. Finally, contrasting hypotheses have been proposed for the content of Sinemyidae^5^,^7^,^10^,^12^,^22^,^26^,^27^,^28^,^29^ pending the inclusion of Pan-Pleurodira in the phylogenetic analysis^13^. With the help of a revised morphological dataset we here reassess the monophyly of Asian and North American Cretaceous basal pan-cryptodire turtles and test between three competing hypotheses that differ by viewing sinemyidids as *(i)* stem-cryptodires, *(ii)* stem-turtles or *(iii)* stem-chelonioid sea turtles.

## Results

### Geological Setting

**Figure 1:**
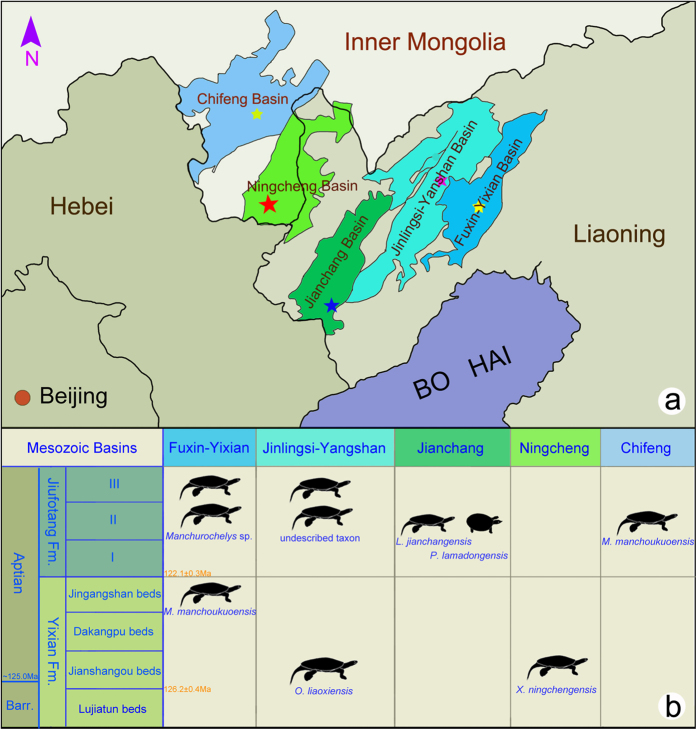
Area map showing the fossil localities and the stratigraphic distribution of turtles in the Mesozoic basins of Inner Mongolia and Liaoning, northeastern China. (**a,b**) The type locality of *Xiaochelys ningchengensis* gen. et sp. nov. (Red asterisk; Yangshuwanzi, Ningcheng County, Chifeng City, Inner Mongolia) and Ningcheng Basin (Light green); *Liaochelys jianchangensis* and *Perochelys lamadongensis* (Blue asterisk; Lamadong, Jianchang County, western Liaoning) and Jianchang Basin (Dark green); *Ordosemys liaoxiensis* (Purple asterisk; Shanyuan, Beipiao City, western Liaoning) and Jinlingsi-Yanshan Basin (Blue green); *Manchurochelys manchoukuoensis* (Yellow asterisk; Jingangshan, Yixian County, western Liaoning; Qilinshan, Chifeng City, Inner Mongolia) and Yixian Basin (dark blue) and Chifeng Basin (light blue). Area map was modified from Zhou *et al.*^13^.

The description of *Xiaochelys ningchengensis* gen. et sp. nov. is based on the holotype and only specimen (PMOL-AR00210) preserved on two slabs. The specimen originates from the Early Cretaceous Yixian Formation (Jehol Biota) of the Ningcheng Basin exposed at Yangshuwanzi Village, Bisiyingzi Township, Ningcheng County, Inner Mongolia Province (Fig. 1). In the Ningcheng Basin, numerous vertebrates are already known from the Middle-Late Jurassic Yanliao Biota, including salamanders, feathered dinosaurs, and early mammals^30^,^31^,^32^. In the younger Jehol Biota, however, tetrapod vertebrate fossils are rare, except for a lamprey *Mesomyzon mengae*, the lizard *Liushusaurus acanthocaudata*, and the pterosaur *Ningchengopterus liuae* from the Yixian Formation of Liutiaogou Village, Dashuangmiao Township, Ningcheng County^33^,^34^,^35^. The new fossil locality is about 18 km south of Liutiaogou Village. Here, the lacustrine deposits of the Yixian Formation consist mainly of mudstone, shale, and sandstone. Associated vertebrates include lycopteriform (*Lycoptera*) and acipenseriform (*Protopsephurus*) fishes, lizards and the stem bird *Confuciusornis*. The fossil-bearing horizon of the Yixian Formation in the Ningcheng Basin is equivalent to the Jianshangou bed (ca. 124–122 Ma) of the Yixian Formation in western Liaoning^36^.

**Systematic Palaeontology.**

**Testudinata Klein, 1760**

**Testudines Batsch, 1788**

**Pan-Cryptodira Joyce, Parham, and Gauthier, 2004**

**Sinemydidae** ***sensu*** **Rabi, Sukhanov, Egorova, Danilov, and Joyce 2014**

***Xiaochelys ningchengensis*** **gen. et sp. nov.**

### Holotype

**Figure 2:**
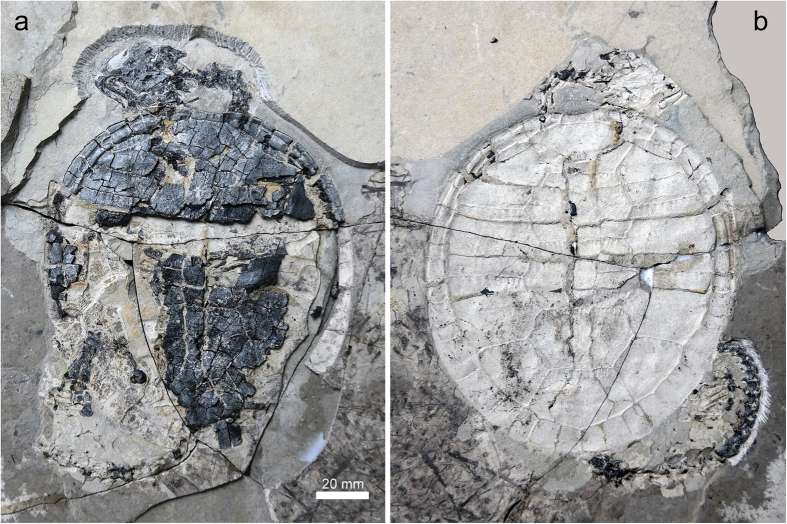
*Xiaochelys ningchengensis* gen. et sp. nov. (PMOL-AR00210AB, holotype) from the Early Cretaceous Jehol Biota (Yixian Formation) of Ningcheng, Chifeng City, Inner Mongolia, northeastern China. (**a,b**) Positive and counter part of the holotype.

**Figure 3:**
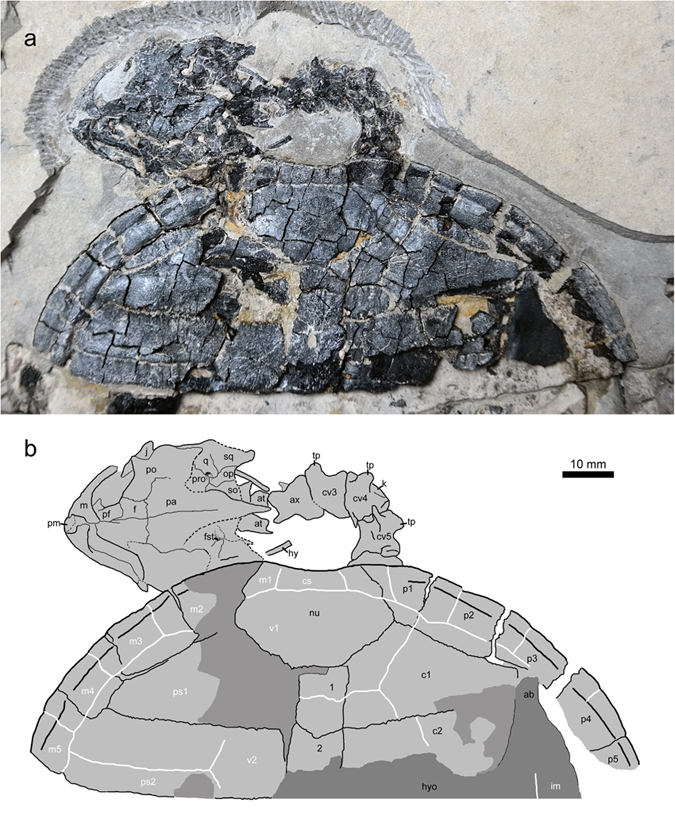
Anterior part of the skeleton of *Xiaochelys ningchengensis* gen. et sp. nov. (PMOL-AR00210A, holotype). Abbreviations: ab, axillary buttress; at, atlas; ax, axis; cs, cervical scale; cv3–cv5, cervical vertebrae 3–5; c1–c2, costal plates 1–2; f, frontal; fst, foramen stapedio-temporale; hy, hyoid; hyo, hyoplastron; im, inframarginal scale; j, jugal; k, ventral keel of the cervical vertebra; m, maxilla; m1–m5, marginal scales 1–5; nu, nuchal; op, opisthotic; pa, parietal; pf, prefrontal; pm, premaxilla; po, postorbital; pro, prootic; ps1–ps2, pleural scales 1–2; p1–p5, peripheral plates 1–5; q, quadrate; so, supraoccipital; sq, squamosal; tp, transverse process of cervical vertebra; v1–v2, vertebral scales 1–2; 1–2, neural plates 1–2.

**Figure 4:**
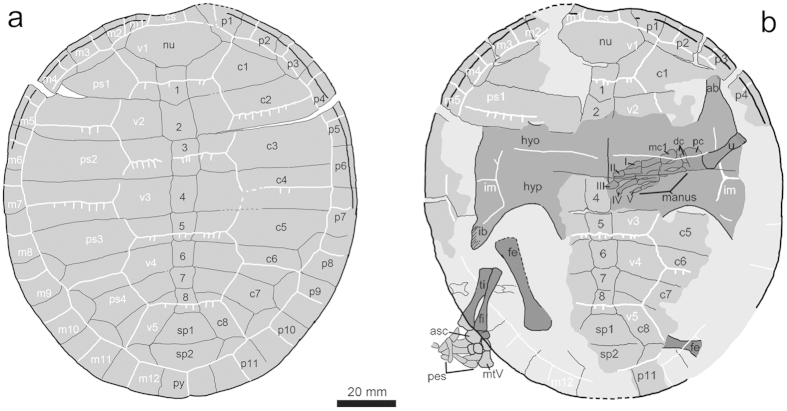
Line drawings of the carapace and plastron of *Xiaochelys ningchengensis* gen. et sp. nov. (PMOL-AR00210AB, holotype). Abbreviations: ab, axillary buttress; asc, astragalocalcaneum; c1–c8, costal plates 1–8; cs, cervical scale; dc, distal carpals; fe, femur; fi, fibula; hyo, hyoplastron; hyp, hypoplastron; I–V, manual digits I–V; ib, inguinal buttress; im, inframarginal scale; m1–m12, marginal scales 1–12; mc1, metacarpal 1; mtV, metatarsal V; nu, nuchal; p1–p11, peripheral plates 1–11; pc, proximal carpals; ps1–ps4, pleural scales 1–4; py, pygal; sp1–sp2, suprapygals 1–2; ti, tibia; u, ulna; v1–v5, vertebral scales 1–5; 1–8, neural plates 1–8.

**Figure 5:**
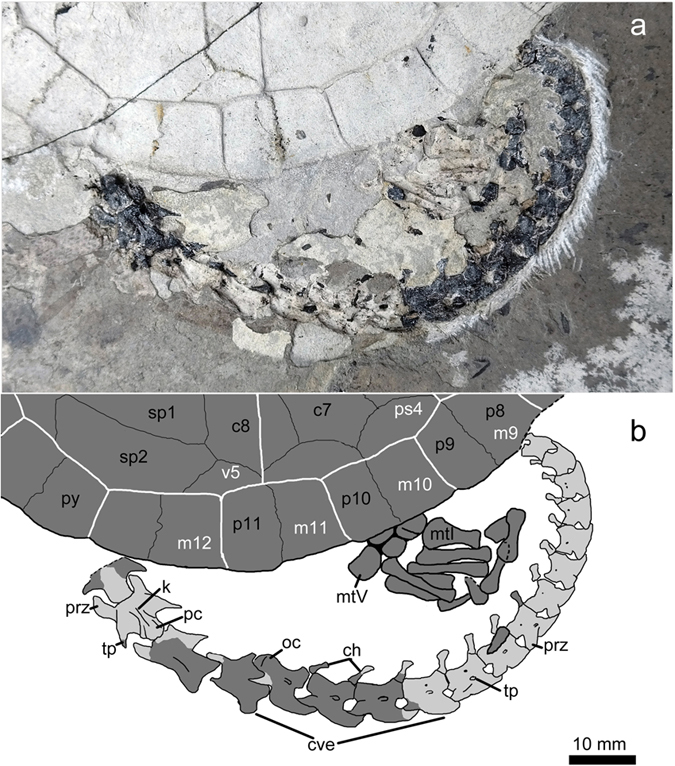
Caudal series of *Xiaochelys ningchengensis* gen. et sp. nov. (PMOL-AR00210B, holotype). Abbreviations: c7–c8, costal plates 7–8; ch, chevrons; cve, caudal vertebrae; k, ventral keel of the caudal vertebra; m9–m12, marginal scales 9–12; mtI–mtV, metatarsals I–V; oc, opisthocoelus vertebra; p8–p11, peripheral plates 8–11; pc, procoelus vertebra; prz, prezygapophysis; ps4, pleural scale 4; py, pygal; sp1–sp2, suprapygals 1–2; tp, transverse process of the caudal vertebra; v5, vertebral scale 5.

Articulated skeleton of a potentially juvenile or subadult individual obtained in two slabs, PMOL-AR00210 (Figs. 2, 3, 4, 5; see Supplementary Note for a discussion on the ontogenetic status of the specimen).

### Type locality and horizon

The fossil site is near to Yang-shu-wan-zi Village, Bi-si-ying-zi Township, Ningcheng County, Chifeng City, Inner Mongolia; Early Cretaceous, Yixian Formation, Barremian-Early–Aptian.

### Etymology

Chinese Pinyin ‘Xiao’, refers to the small size of the taxon, ‘chelys’ Greek ‘turtle’; the specific epithet refers to the type locality.

### Diagnosis

*Xiaochelys ningchengensis* differs from other Cretaceous sinemydid and macrobaenid turtles by its unusual combination of features: midline contact of prefrontals present; supraoccipital crest longer than squamosal horn; fourth cervical biconvex; nuchal emargination shallow; cervical scale present; first vertebral wider than nuchal; first vertebral overlapping second peripheral; first vertebral contacting second marginal; vertebral and pleural sulcus plications present; preneural absent; the presence of eight neurals, two subequal suprapygals, and a pygal; third costals with parallel anterior and posterior sides; costo-peripheral, lateral and central plastral fontanelles absent.

### Comparative description

The skull is exposed in dorsal view (Fig. 3). The upper temporal emargination is developed but its anterior rim is uncertain. It appears to be comparable with that of *Ordosemys* spp. The crista supraoccipitalis extends slightly beyond the distal level of the squamosals but not to the degree of *Manchurochelys manchoukuoensis*^19^.

The premaxilla forms the ventral rim of the apertura narium externa. Under the fossa orbitalis, the horizontal (palatal) portion of the maxilla is partially exposed and reveals a narrow triturating surface. The nasals, if present, are not exposed. The dorsal plate of the prefrontal is a large element that forms the interorbital roof. Anteriorly, the prefrontal has a midline contact with the other prefrontal, different from the separated condition seen, for instance, in *Ordosemys* spp.^18^,^21^,^27^. Posteriorly, the prefrontals are partially separated by the insertion of the frontal processes. The frontal is situated between the prefrontal anteriorly, postorbital laterally, and parietal posteriorly. Combined with the latter two elements, the frontal forms the dorsal and posterior rim of the orbit. Laterally, the parietal has a long suture with the postorbital and forms the anterior rim of the upper temporal emargination with the latter.

Anteriorly, the jugal has a slender process to contact the maxilla under the ventral rim of the orbit. Dorsally, the jugal has a curved contact with the postorbital. Other posterior contacts of the jugal are uncertain due to compression. The postorbital is the largest element in the temporal region delimiting the deep upper temporal emargination together with the parietal. Laterally, it has a curved margin, possibly for articulation with the jugal. Posteriorly, the postorbital contacts the squamosal. The squamosal bears a low dorsal ridge that extends longitudinally to frame the lateral aspects of the upper temporal emargination. The posteriormost tip of the squamosal is pinched and directed posterolaterally as in *M*. *manchoukuoensis* and *Kirgizemys hoburensis*. Medial to the ridge, the squamosal contacts the quadrate anteriorly and the opisthotic posteromedially. Due to the deep upper temporal emargination, the otic capsule is exposed in dorsal view. The foramen stapedio-temporale is large and formed by the quadrate, prootic, opisthotic and supraoccipital. The supraoccipital crest is distally damaged in the positive slab, but its imprint in the negative slab clearly indicates that the crest is longer than those of *O. liaoxiensis* or *L. jianchangensis* but shorter compared to that of *M*. *manchoukuoensis*.

The carapace of PMOL-AR00210 is oval and flat (Figs. 2 and 4). It is about 130.4 mm long along the midline with a maximum width of about 119.6 mm at the level of the fourth costal plates. The carapacial surface is generally smooth with the exception of some plications that decorate the vertebrals and pleurals. A longitudinal midline depression is absent in contrast to *M. manchoukuoensis*, *Ordosemys leios*, *O*. *liaoxiensis*, *Liaochelys jianchangensis*^16^,^18^,^19^,^21^.

The nuchal forms a shallow emargination, as in *M*. *manchoukuoensis*, and *L*. *jianchangensis* but different from *Changmachelys bohlini* and *Ordosemys* spp., where the emargination is more developed^18^,^37^. The nuchal contacts the first peripherals laterally, the first costals posterolaterally, and the first neural posteriorly. In contrast, the nuchal is laterally more expanded and has an additional contact with the second peripherals as in *Ordosemys leios* and *O*. *liaoxiensis*^18^,^21^. The neural series is composed of eight elements. A preneural, diagnostic for the genus *Ordosemys*, is notably absent. Anterior neurals are subrectangular but the last three neurals are varied in shape: neurals 6–7 are hexagonal with short anterolateral sides and neural 8 is subtrapezoidal with a longer posterior side. Neural 2 is slightly larger than neural 1 and the following neurals decrease in size posteriorly, reaching their minimum size at neural 7. Two trapezoidal suprapygals are present. Suprapygal 1 is slightly narrower than suprapygal 2, different from the reduced suprapygal 1 seen in *M*. *manchoukuoensis*, and *L*. *jianchangensis*. Suprapygal 1 is separated from the peripherals by a contact between suprapygal 2 and costals 8, as in *M*. *manchoukuoensis* and *L*. *jianchangensis*, and *O*. *liaoxiensis*. Distally, the suprapygal-pygal suture is confluent with the sulci of vertebral and marginal scales. A pygal element is present between peripherals 11 and is comparable with them in size. The pygal has a shallow posterior midline emargination.

The first two pairs of costals are preserved as actual bone whereras the rest is visible in form of imprints in the negative slab. Costo-peripheral fontanelles are notably closed in PMOL-AR00210 unlike in taxa with larger adult sizes, including *O. liaoxiensis* and *C. bohlini*^18^,^37^. The first costal plate has divergent anterior and posterior sides, as in *M*. *manchoukuoensis*, and different from the parallel sided condition seen in *L*. *jianchangensis*, *Ordosemys* spp., and *C*. *bohlini*. Costal 1 has a broad contact with peripherals 1–3 as in *M*. *manchoukuoensis* and unlike in *Ordosemys* spp. (with the peripherals 2–3) and *L*. *jianchangensis* (with 1–4)^16^,^18^,^19^,^21^. Costal 2 is subrectangular in shape. It is wider than the first costal but much shorter anteroposteriorly. The anterior and posterior sides of costal 2 are parallel, different from the distally converging sides apparent in *L*. *jianchangensis*^16^. Distally, costal 2 contacts peripherals 3–4 as in *Ordosemys* spp. but unlike in *L*. *jianchangensis* (contacts peripheral 4 only) or *M*. *manchoukuoensis* (contacts peripherals 3–5)^16^,^18^,^19^,^21^. Costal 3 is the largest costal plate with parallel anterior and posterior sides, as in *Ordosemys* spp. and *M*. *manchoukuoensis*, contrasting with the distally-expanded condition unique for *L*. *jianchangensis*. Distally, costal 3 is wedged between peripherals 5–6. Costal 4 is comparable with costal 3 in size and wedges distally between peripherals 6–7. As apparent from the imprint of the carapace, the distal ends of the posterior costals are narrow and bordered by the peripherals in dorsal view as in *Ordosemys liaoxiensis*, *L*. *jianchangensis*, *Sinemys* spp., *M*. *manchoukuoensis* and *Dracochelys biscupis*^16^,^18^,^19^,^38^. It remains unclear whether the rib ends were exposed.

The peripherals (eleven pairs) are guttered from peripheral 1 to 7. Peripheral 1 is subtriangular in shape and contacts the nuchal plate medially, peripheral 2 laterally, and costal 1 posteriorly. The peripheral 1-costal 1 contact is absent or very short in *O*. *leios*, *O*. *liaoxiensis*, and *D*. *bicuspis*^18^,^21^,^38^. Peripheral 2 is enlarged and has a rectangular outline. Peripheral 3 is reduced relative to peripheral 2 and receives the rib end of the first costal plate. Peripherals 8–11 are expanded and covered by pleural sulci as in most other basal eucryptodires but unlike in *Macrobaena mongolica* where the pleural scales barely reach the peripherals.

Marked sulcus plications extend posteriorly from the transversely oriented sulci of the vertebral and pleural scales. The cervical scale is slender, wider than long, but much narrower than the nuchal emargination. The vertebrals are wider than long and also wider than the pleurals. Vertebral 1 is wider than the nuchal and barely overlaps onto peripheral 2. In contrast, vertebral 1 barely touches even peripheral 1 in *O. leios*, *O*. *liaoxiensis*, *C. bohlini*, *L. jianchangensis*, and *M. manchoukuoensis*. Vertebrals 2–4 are hexagonal and vertebral 2 is slightly wider and considerably longer than vertebral 1. The vertebral 1–2 sulcus has a small anterior midline projection across the midlength of neural 1. Vertebral 3 is the widest element with subequal anterior and posterior lateral sides. The vertebral 3–4 sulcus extends onto the posterior portion of neural 5. Vertebral 5 is pentagonal in outline, larger than the suprapygals and extends onto peripheral 11 laterally. Pleurals 1–3 are wider than long whereas pleural 4 is longer than wide. Marginal 2 has a broad contact with vertebral 1, as in *M*. *manchoukuoensis* and *L*. *jianchangensis* and unlike the point-like contact seen in *O*. *leios* and *O*. *liaoxiensis*^18^,^21^. The pleuro-marginal sulci of marginals 5–7 coincide with the costo-peripheral suture. Marginals 12 overlap the pygal.

Of the plastron large parts of the hyo- and hypoplastra are preserved as bone or as imprints (Fig. 4b). The plastron connected to the carapace via ligaments and pegs. The hypoplastron is anteroposteriorly short at the level of the inguinal notch with a short distance between the inguinal and axillary notches unlike the long bridge of *Manchurochelys manchoukuoensis* and *Sinemys* spp. but similarly to most other Cretaceous basal eucryptodires from Asia. There is no trace of a central or lateral fontenelles in the imprint of the plastron, similarly to *K. hoburensis*. Inframarginals are present but their number is uncertain. The axillary buttress is partially exposed on the right side and seems to contact peripheral 3 distally or even beyond. Posteriorly, the inguinal buttress terminates on peripheral 8.

Of the vertebral column only the cervical and caudal vertebrae are exposed. The anterior five cervical vertebrae are in articulation. Some fragments of the series are exposed in the counterpart slab. The atlas is disarticulated with two neural arches and a small and subrectangular centrum. The atlas neural arch is a flat lamina and is clearly shorter than the axis. Posterolaterally, it possesses a short spine. The axis is comparable with the remaining three exposed cervicals in size. The centrum of cervical 4 appears to be biconvex as in *O. leios* and *K. hoburensis*, whereas the fifth centrum is procoelous. A ventral keel is exposed along cervical 4. The transverse process is anteriorly positioned near the distal end of the centrum. No cervical ribs are apparent but the posterior vertebrae may have possessed reduced ribs in *X*. *ningchengensis* as in other stem-cryptodires (e.g. *L. jianchangensis*).

18 articulated caudal vertebrae can be identified on the negative slab (Fig. 5) either as bones or as imprints over a total length of about 12 cm. At least two more proximal caudals and six more distal caudals are visible through a broken region of the carapace in the positive slab. The distal end of the tail is incomplete. In the negative slab, the anterior two caudals are exposed in ventral view and show procoelous centra and weekly-developed ventral keels. The prezygapophyses are well developed and extend anteriorly well beyond the centra. The centrum of the fifth preserved caudal appears to be opisthocoelous, implying the presence of a caudal transition from anteriorly procoelous to posteriorly opisthocoelous caudals, as in *O. leios*, *O*. *liaoxiensis*, and *Judithemys sukhanovi*. Moreover, the fourth preserved caudal shows an opisthocoelous centrum in the positive slab, implying that the third preserved caudal is biconcave. Considering the two more anterior caudals preserved in the positive slab under the shell, the transitional caudal is possibly the anatomical fifth or even more distal. This is comparable to *J*. *sukhanovi*, where the sixth caudal is the transitional vertebra^7^. A more proximal condition is present in *Ordosemys* spp. and *Sinemys brevispinus*, in which caudal 3 or 4 is biconcave^18^,^21^,^39^. The centrum of the posterior caudals is strongly constricted bilaterally and has a distinctly curved ventral margin. Along the posterior caudal series, one or two foramina are present on the lateral surface of the centrum. The transverse processes of the caudal centra are posterolaterally projecting, strongly reduced in size posteriorly along the series and disappear by the eighth exposed caudal in the negative slab. Chevra are distinct along the series, strongly articulate with adjacent caudals and decrease in size posteriorly. The chevrons are Y-shaped with an expanded distal end.

The appendicular skeleton is partially preserved on the positive slab and preserved either as imprints or bones. The right forelimb (ulna and manus) and the left hind limb (femur, tibia, fibula, and pes) are preserved the best (Figs. 2 and 4b). The imprint of the right ulna reveals that it was slender with an expanded distal end. The manus is relatively elongated, nearly twice the ulnar length, as in *C. bohlini*, likely implying a highly aquatic lifestyle^40^. The carpus is well ossified and its elements are firmly articulated via movable joints. The proximal carpals are large sized and form a pronounced block between the distal carpals and the ulna. In contrast, the three distal carpals exposed are small. They are arranged against the metacarpals I-III. Metacarpal I is short and robust, metacarpal II is elongated and slender and metacarpal III is the longest and narrowest. The manual digits are preserved in articulation. The phalangeal formula was likely 2-3-3-3-3, although the ungual of digit IV is missing and the proximal portion of digit V is covered by the hypoplastron. Digit I is short and robust, digit II is elongated and digit III is the longest one. Digit V is slender and bears a reduced ungual.

The tibia and fibula are subequal in length and considerably shorter than the femur. Proximally, the tibia and the fibula articulate with one another along a long contact whereas their distal articulation is much reduced. The tarsals and metatarsals are preserved in articulation whereas the phalanges are disarticulated. The astragalus is possibly fused together with the calcaneum, forming a large block against the fibula. Metatarsal I is robust with an expanded proximal end. Metatarsls II-IV are elongated and slender. The ansula is large and hook-like. The pedal formula is uncertain.

### Phylogenetic relationships

**Figure 6:**
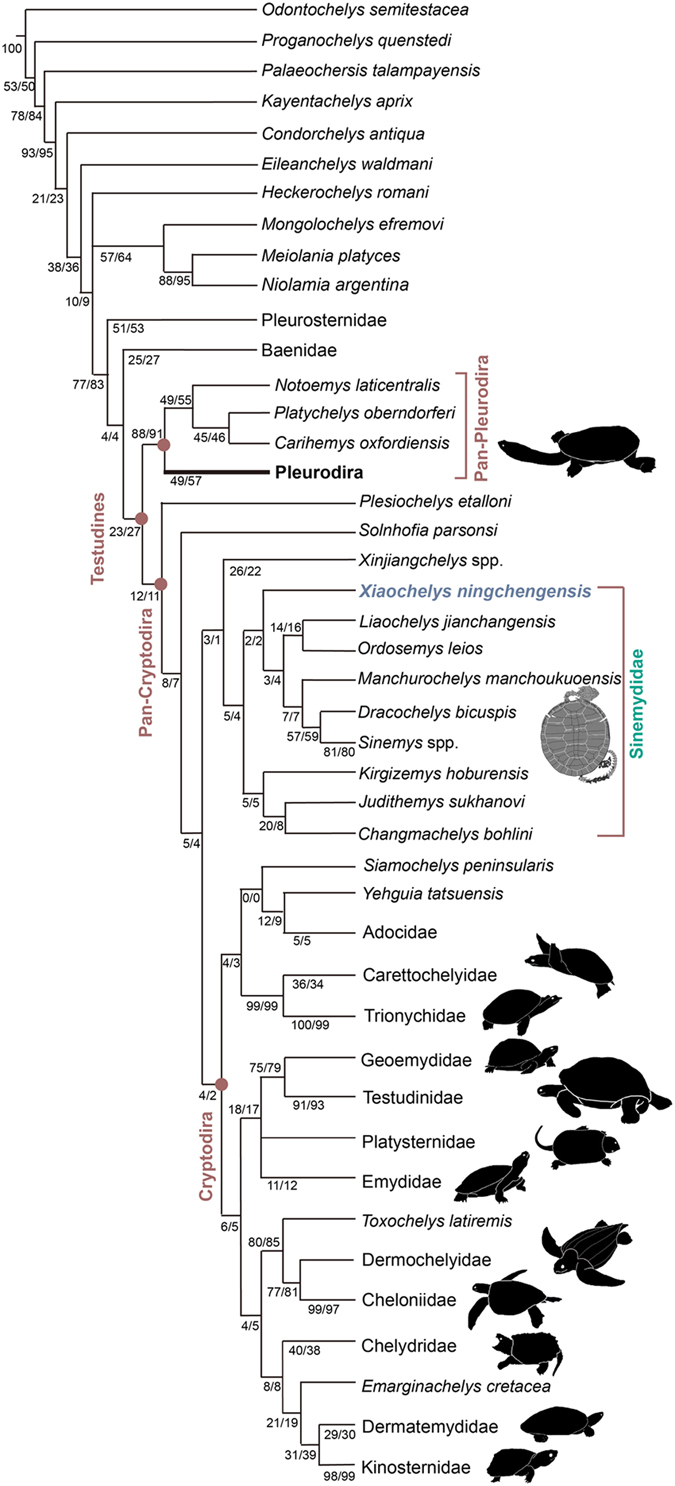
Simplified strict consensus tree of the global turtle phylogenetic analysis employing a molecular scaffold^25^. *Xiaochelys ningchengensis* gen. et sp. nov. is a member of Sinemydidae, a poorly supported clade that also includes *Changmachelys bohlini*, *Kirgizemys hoburensis* and the North American Late Cretaceous *Judithemys sukhanovi* following the phylogenetic definition of Rabi *et al.*^20^. Together with xinjiangchelyids, none of these taxa are retrieved as stem-turtles or stem-chelonioids in contrast to several earlier studies. Numbers correspond to Bootstrap/Jackknife values.

Parsimony analysis of the dataset retrieved 1469 trees of 854 steps (CI = 0.353; RI = 0.801; see Supplementary Data). *Xiaochelys ningchengensis* is placed as a member of Sinemydidae (as defined by Rabi *et al.*^20^), a poorly supported clade that furthermore includes *Changmachelys bohlini*, *Judithemys sukhanovi*, *Kirgizemys hoburensis*, *Liaochelys jianchangensis*, *Ordosemys leios*, and a clade consisting of *Manchurochelys manchoukuoensis*, *Dracochelys bicuspis* and *Sinemys* spp. (Fig. 6). The placement of *X*. *ningchengensis* in Sinemydidae is supported by the exposure of the posterior costal rib ends within the peripherals (Costal rib distal end; a synapomorphy of Sinemydidae in all of the trees; see common synapomorphies in Supplementary Information). Sinemydidae forms a monophyletic clade with Xinjiangchelyidae on the stem of Cryptodira.

## Discussion

### Turtle diversity of the Jehol Biota

Four taxa of turtles have been described from the type area of the Jehol Biota (the conjoint area expanding from Liaoning Province to Inner Mongolia Province) including *Manchurochelys manchoukuoensis*, *Ordosemys liaoxiensis* and *Liaochelys jianchangensis*, and the soft-shelled turtle *Perochelys lamadongensis*. The former three taxa belong to Sinemydidae based on our phylogenetic analysis and the definition of Rabi *et al.*^20^ (but note that *Ordosemys* is represented by *leios* in our matrix, not *liaoxiensis*). Turtles of the Jehol Biota appear to show a complex palaeobiogeographic and stratigraphic distribution, especially when formally yet undescribed taxa and records are also considered (Fig. 1). For example, *M*. *manchoukuoensis* is the only taxon known so far from both the Yixian Formation of the Fuxin-Yixian Basin and the Jiufotang Formation in Chifeng Basin^13^,^19^ but undescribed PMOL specimens of *Manchurochelys* sp. may confirm the presence of this taxon in the Jiufotang Formation of the Fuxin-Yixian Basin as well. *Ordosemys liaoxiensis* is known from the Yixian Formation of the Jinlingsi-Yanshan Basin^15^,^18^ but undescribed PMOL specimens are also known from Jiufotang Formation of the same basin (Fig. 1). *L*. *jianchangensis* and *P*. *lamadongensis* both originate from the Jiufotang Formation of the Jianchang Basin (Fig. 1)^16^,^17^. *Xiaochelys ningchengensis* from the Yixian Formation of the Ningcheng Basin further increases the complexity of Jehol Biota turtles in terms of taxonomic diversity and spatiotemporal distribution.

### The basal divergence of crown Testudines

One of the most notable results of our analysis is the placement of xinjiangchelyids and sinemydids along the stem lineage of crown Cryptodira. Recent analyses^12^,^13^,^20^ have placed these taxa outside of the crown-group of turtles in contrast with traditional systematic viewpoints as well as earlier phylogenies^2^,^4^,^6^,^7^,^8^,^9^,^26^,^28^,^41^. This unorthodox signal is due to the unstable position of Pleurodira which has been an issue since the morphologic phylogeny of Joyce^8^ where Pleurodira nested within Cryptodira when characters were treated unordered. Even a number of molecular and total evidence analyses have failed to overcome this issue and retrieved a paraphyletic Cryptodira with a deeply nested Pleurodira^23^,^42^,^43^. On the other hand, the far majority of existing phylogenies, including the most comprehensive recent molecular study, strongly demonstrate the monophyly of both Cryptodira and Pleurodira^4^,^8^,^9^,^22^,^25^,^28^,^42^,^44^,^45^,^46^,^47^,^48^.

One reason for the contradictory placement of Xinjiangchelyidae, Sinemydidae and similar taxa outside the crown is the recent identification of primitive morphologies in these taxa, such as the retention of nasals and a pair of basipterygoid processes, partially open carotid circulation, an interpterygoid slit, long dorsal rib 1, rudimentary epiplastral processes, cervical ribs, and the absence of a fully derived “cryptodiran neck”. The simplification and reinforcement of both the skull and shell is widely accepted to have happened in parallel between cryptodires and pleurodires^8^,^12^,^24^,^44^,^46^. However, while data on stem-cryptodire morphology has substantially increased in the last twenty years our knowledge on the pleurodire stem-lineage remained insufficient^49^,^50^,^51^. In recent phylogenies^12^,^13^,^20^, this resulted in the optimization of characters pertaining to the reinforcement of the skull and shell as synapomorphies for a clade of extant pleurodires and cryptodires only and to the exclusion of the many taxa traditionally placed on the cryptodiran stem.

Since the character sample of recent global morphologic matrices^8^,^9^,^11^,^12^,^13^,^19^,^20^,^22^,^23^,^28^,^41^,^47^,^48^,^52^ not fully considered the carotid circulation pattern of sinemydids we expanded the sample and redefined the pertaining characters of previous works (see Supplementary Data). A parsimony analysis of this dataset corroborated the conventional placement of Xinjiangchelyidae and Sinemydidae on the stem of Cryptodira. Four additional steps are required to place these taxa on the stem of the turtle-crown. This result further indicates that the characters pertaining to the reinforcement of the skeleton in crown cryptodires and crown pleurodires are homoplastic.

### Could sinemydids be stem-chelonioid sea turtles ? 

The origin of chelonioid sea turtles is rather cryptic and the sequence of skeletal adaptations associated with the transition from freshwater to marine environment is poorly understood likewise. Turtles traditionally referred to Sinemydidae and/or Macrobaenidae have been often hypothesized to form the stem lineage of chelonioid sea turtles, or at least noted to be phenetically similar to marine turtles^5^,^21^,^22^,^23^,^25^,^52^. Indeed, the primitive pan-chelonioid *Toxochelys latiremis*^53^ greatly resembles *Judithemys* spp. and some sinemydids in gross cranial and shell morphology (e.g. reduction of carapace and plastron). The historical, though erroneous assignment of postcranial material of North American macrobaenids to the primitive pan-chelonioid *Osteopygis* spp^54^. illustrates this point well. However, most studies placing sinemydids and macrobaenids as stem-chelonioids employed no molecular framework (except Danilov and Parham^22^).

**Figure 7:**
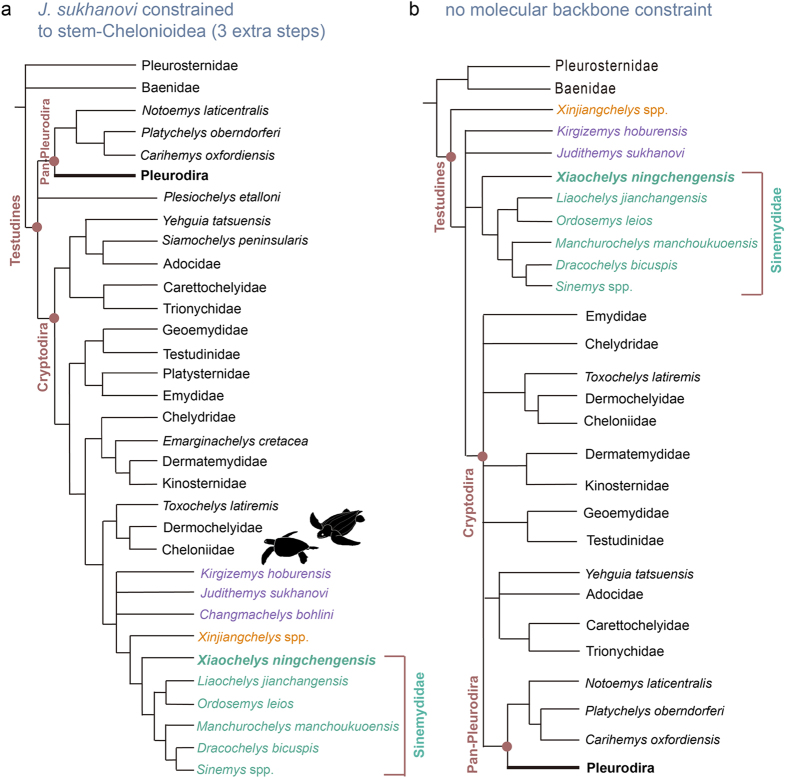
(**a**) Effect on topology and tree length of forcing *Judithemys sukhanovi* to the stem of Chelonioidea. This analysis also implemented a molecular backbone. (**b**) Reduced strict consensus tree of parsimony analysis employing no molecular backbone constraint. Green colour denotes Sinemydidae, orange Xinjiangchelyidae sensu Rabi *et al*.^20^ and blue Macrobaenidae sensu Brinkman *et al.*^37^.

We tested the hypothesized content of the sea turtle stem by forcing *Judithemys sukhanovi* the taxon that had repeatedly been found closer to the cryptodiran crown^5^,^7^,^9^,^10^,^22^,^26^,^28^,^37^, to the stem of Pan-Chelonioidea, and setting all other sinemydids, xinjiangchelyids, *Siamochelys peninsularis*, *Yehguia tatsuensis*, *Solnhofia parsonsi* and *Plesiochelys etalloni* as floaters (i.e. they can float within the constraints following the search). In the resulting pruned consensus tree (Fig. 7a), *J. sukhanovi* pulled xinjiangchelyids, sinemydids, and in some trees *Solnhofia parsonsi* into crown Cryptodira to form an extended stem linage of Pan-Chelonioidea. Notably, the resulting trees (n = 329) are only three steps longer (n = 857) than the trees interpreted these taxa as stem-cryptodires. Four synapomorphies support the pan-chelolonioid placement in the majority of the trees: a moderate upper temporal emargination, the contribution of the pterygoid to the foramen palatinum posterius, the loss of a double articulation between the 6^th^ and 7^th^ cervicals, and the reversal to an anteriorly facing 1^st^ dorsal vertebra. These synapomorphies are either not unique to this clade or consist of reversals to the primitive condition and therefore does not provide strong evidence against the more parsimonious stem-cryptodire hypothesis. Moreover, *J. sukhanovi* pulls all other Asian stem-cryptodires to the stem of sea-turtles including the Jurassic xinjiangchelyids. This would infer a Jurassic age for the origin of chelydroids and testudinoids which is in contrast with the fossil record and recent molecular divergence date estimates of cryptodire clades^55^. Forcing *J. sukhanovi* to the stem of Trionychia, Durocryptodira, Testudinoidea, Americhelydia, Chelydroidea, Kinosternoidea or Chelydridae results in at least six steps longer trees (see Supplementary Discussion). This likely indicates that among crown-cryptodires, pan-chelonioids share the most symplesiomorphies with stem-cryptodires.

## Conclusions

*Xiaochelys ningchengensis* adds to the diversity of turtles of the Jehol Biota. The type and only specimen may represent a subadult or a juvenile individual but it possesses enough characters to differentiate it from other, similar taxa. Moreover, a parsimony analysis does not place it particularly close to any other genera.

The new phylogeny presented herein confutes recent tentative hypotheses that consider Sinemydidae and closely allied taxa as stem-turtles. Instead, cryptodires had a diverse stem-lineage of Jurassic and Cretaceous taxa in Asia and North America including at least xinjiangchelyids and sinemydids. Our analysis therefore confirms that skeletal reinforcement evolved independently in crown pleurodires and cryptodires.

Contrary to the results of earlier studies^5^,^6^,^7^,^9^,^10^,^12^,^13^,^22^,^26^,^28^,^29^, all Early Cretaceous and Jurassic stem-cryptodires from Asia form a monophyletic clade in our analysis which corresponds to the Sinemydidae and Xinjiangchelyidae of Rabi *et al.*^20^. Judging from the low statistical support of this clade (Fig. 6), however, further studies could still demonstrate the paraphyletic arrangement of the cryptodiran stem. *Kirgizemys hoburensis*, *Changmachelys bohlini* and the North American Late Cretaceous *Judithemys sukhanovi* are here retrieved as basal sinemydids but the inclusion of *Macrobaena mongolica* may call for their re-classification as macrobaenids in the future.

Much of the great phenetic similarities between chelonioid sea turtles and sinemydids are ancestral characters and placing these taxa on the stem of Chelonioidea is not the most parsimonious hypothesis. Our study therefore does not support the hypothesis in which sinemydids, *J. sukhanovi* or *K. hoburensis* are regarded as sea-turtle ancestors. However, since character support against the stem-chelonioid hypothesis is minor and little is known about character evolution at the base of Cryptodira, Chelonioidea, Amerchelydia and Durocryptodira, future discoveries may change the placement of a few taxa that are here retrieved as sinemydids. Our results are nevertheless consistent with the post-Jurassic (Early Cretaceous) molecular divergence date estimate for the origin of pan-chelonioid sea turtles^55^.

We present the most up to date global phylogeny of extinct turtles which solves a major problem emerging in recent morphologic studies; the unorthodox placement of Pan-Pleurodira. We therefore suggest using this matrix as a framework and starting point for future analyses of extinct turtle relationships. In addition to the fact that the unconstrained morphological phylogeny places pan-pleurodires in an unconventional position (Fig. 7b) and is inconsistent with the stratigraphic distribution of turtles^25^ we point out that most studies dubiously retrieving sinemydids, *J. sukhanovi* and *K. hoburensis* as stem-chelonioids did not employ a molecular backbone constraint. We therefore further emphasize the necessity of using a molecular scaffold in global turtle morphological analyses^25^.

## Methods

Anatomical comparisons were made based on direct observation (M.R. & C.-F.Z.) of all relevant Cretaceous basal eucryptodire taxa (Supplementary Materials). Taxonomic nomenclature follows Danilov and Parham^26^, Joyce *et al.*^55^,^56^ and Rabi *et al.*^20^.

A modified version of the character matrix of Zhou *et al.*^13^ was used for the phylogenetic analysis (see Related Manuscript File: Taxon_character matrix.txt). Given that this analysis is focused on the phylogenetic relationships and placement of sinemydid, xinjiangchelyid and other similar turtles, we decided to crop taxa not pertinent to these questions and a broad spectrum of taxa known from fragmentary material only in order to reduce the size of the matrix (Supplementary Data). 7 new characters were added and changes in the scorings were introduced in the matrix (Supplementary Data). The resulting matrix consists of 245 characters for a total of 87 terminal taxa (Supplementary Data).

A heuristic search was performed on the dataset in TNT^57^,^58^ using the tree-bisection-reconnection swapping algorithm with 1000 random addition sequence replicates and 10 trees saved per replicate. A molecular backbone constraint was implemented in the search following Danilov and Parham^22^, Rabi *et al.*^12^,^20^, Crawford *et al.*^25^ by forcing the relationships of cryptodires to the molecular topology^25^,^43^ (see Supplementary Data). Standard bootstrap and Jacknife values were calculated with 1000 replicates.

## Additional Information

**How to cite this article**: Zhou, C.-F. and Rabi, M. A sinemydid turtle from the Jehol Biota provides insights into the basal divergence of crown turtles. *Sci. Rep.* **5**, 16299; doi: 10.1038/srep16299 (2015).

## Supplementary Material

Supplementary Information
