# Supplementary material for: A sinemydid turtle from the Jehol Biota provides insights into the basal divergence of crown turtles
Source: Sci Rep. 2015 Nov 10;5:16299. doi: 10.1038/srep16299 (PMC4639786; doi:10.1038/srep16299)
Supplement: Supplementary Information [file srep16299-s1.pdf]

Supplementary Information

A sinemydid turtle from the Jehol Biota provides insights into the basal divergence of crown turtles

Chang-Fu Zhou[1*], and Márton Rabi[2,3*]

[1]Paleontological Institute, Shenyang Normal University, Shenyang 110034, Liaoning, China;

[2]Institut für Geowissenschaften, University of Tübingen, Hölderlinstraße 12, 72074 Tübingen, Germany. E-mail: iszkenderun@gmail.com

[3]Department of Paleontology & MTA – ELTE Lendület Dinosaur Research Group, Eötvös Loránd University, Budapest, Hungary.

Correspondence and requests for materials should be addressed to C.-F.Z. (e-mail: zhoucf528@163.com) or M. R. (e-mail: iszkenderun@gmail.com).

Content:

Supplementary Note

Supplementary Discussion

Supplementary Materials

Institutional abbreviations

Supplementary Data

Supplementary References

Synapomorphy Tree

Supplementary Note

Ontogeny and taxonomy. *Xiaochelys ningchengensis* is known from a single and admittedly small-sized specimen (130.4 mm maximal carapace length) therefore its ontogenetic stage needs to be clarified. Moreover, it has a circular carapace and possesses wide vertebral scales, often characteristic of juvenile turtles. However, juvenile turtles almost exclusively have a poorly ossified shell with fontanelles between the costals and peripherals and in the central and bridge area of the plastron. *X*. *ningchengensis* had a fully ossified shell however with all fontanelles closed. Although few growth series have been reported for fossil turtles, a notable exception to the above mentioned pattern happens to include another Chinese turtle from the Early Cretaceous: *Sinemys lens* Wiman 1930 (Brinkman and Peng 1993a). All individuals of *S. lens* come from a single formation and, though greatly differ in size, all have a fully ossified carapace (the plastron is often unknown). These individuals are interpreted to represent an ontogenetic series (Brinkman and Peng 1993a). The smallest individual of *S*. *lens* has a circular, well-ossified carapace, wide vertebrals and a well-developed spine on peripheral 7 whereas in successively larger individuals the carapace becomes gradually more elongated with narrower vertebrals and a reduced peripheral spine. A negative allometric relationship between body size and neural spine length has been reported for the extant emydid *Graptemys* spp. (Pritchard 1979). Thus *S. lens* potentially provides an example of full ossification of the carapace during a juvenile ontogenetic stage and raises the possibility that the only specimen of *X*. *ningchengensis* is a juvenile, especially given that it is also considered a sinemydid. However, *X. ningchengensis* exhibits enough non-ontogeny-related

morphological differences that warrant the introduction of a new taxon. *X. ningchengensis* is distinct from other sinemydids, including *Manchurochelys manchoukuoensis* in having a moderate upper temporal emargination, larger neural 8, and a larger first suprapygal; *Dracochelys biscupis* in having a shallow nuchal emargination, eight neurals, closed costo-peripheral and central-lateral plastral fontanelles even in subadults, upturned peripherals; *Liaochelys jianchangensis* and *Ordosemys* spp*.* in having a longer supraoccipital crest, closed plastral fenestrae, and lacking a distally-expanded costal 3 (*L*. *jianchangensis*) or a preneural plate (*Ordosemys* spp.); and *Sinemys* spp. in having a long postorbital, presence of a pygal and cervical scale, and lacking the lateral spine on peripheral 7 and the central and lateral plastral fontanelles.

Moreover, *X*. *ningchengensis* is excluded from *Wuguia* spp. by a narrower nuchal and the absence of additional ossifications in the nuchal and suprapygal region; and from *Changmachely bohlini* by closed costo-peripheral fontanelles in small size, and closed plastral fenestrae. It differs from *Kirgizemys* (=*Hangaiemys*) spp., *Judithemys sukhanovi*, and *Macrobaena mongolica* in having a narrower distal end of posterior costals. *X*. *ningchengensis* is furthermore different from *Macrobaena mongolica* (PIN 533-4) in having a narrow interorbital roof, pleural sulci extending on peripherals 8-11, and foraminae on the lateral sides of the caudal centra; and from *Judithemys sukhanovi* in having upturned peripherals and subrectangular anterior neurals.

Given that *X*. *ningchengensis* can not be referred to any known genus or species with confidence we here recognize it as a separate taxon. This is also supported by our

phylogenetic analysis that places it as the sister taxon of a larger clade of sinemydids. Future discoveries may clarify the ontogenetic stage of the type specimen and may revise its taxonomic status accordingly.

Supplementary Discussion

The effect on topology and tree length of forcing *Judithemys sukhanovi* to various crown-cryptodire clades

To reveal how tree length changes under different hypotheses where sinemydids are placed on the stem of various cryptodiran clades, we conducted multiple analyses in which *Judithemys sukhanovi* (a member of Sinemydidae in our analysis but has been repeatedly found closer to the cryptodiran crown) was forced to the stem of Chelydroidea, Kinosternoidea, Americhelydia, Durocryptodira, Testudinoidea and Trionychia, respectively. All analyses included a molecular backbone and fossil taxa were set as floaters (see list above; except *J. sukhanovi*). Forcing *Judithemys sukhanovi* is a more conservative approach for testing the robustness of the placement of sinemydids than forcing other sinemydids.

Constraining *J. sukhanovi* to the stem of Chelydroidea yields 206 equally parsimonius trees of 860 steps and a consensus where xinjiangchelyids, sinemydids, *Changmachelys bohlini,* and *Kirgizemys hoburensis* float into crown-cryptodira and placed as stem-chelydroids (Fig. S2). The most parsimonious trees are six steps longer than that of the unforced analysis. One synapomorphy supports this clade: a tail as long as the carapace which is otherwise optimized as a symplesiomorphies for Cryptodira in the unforced analysis.

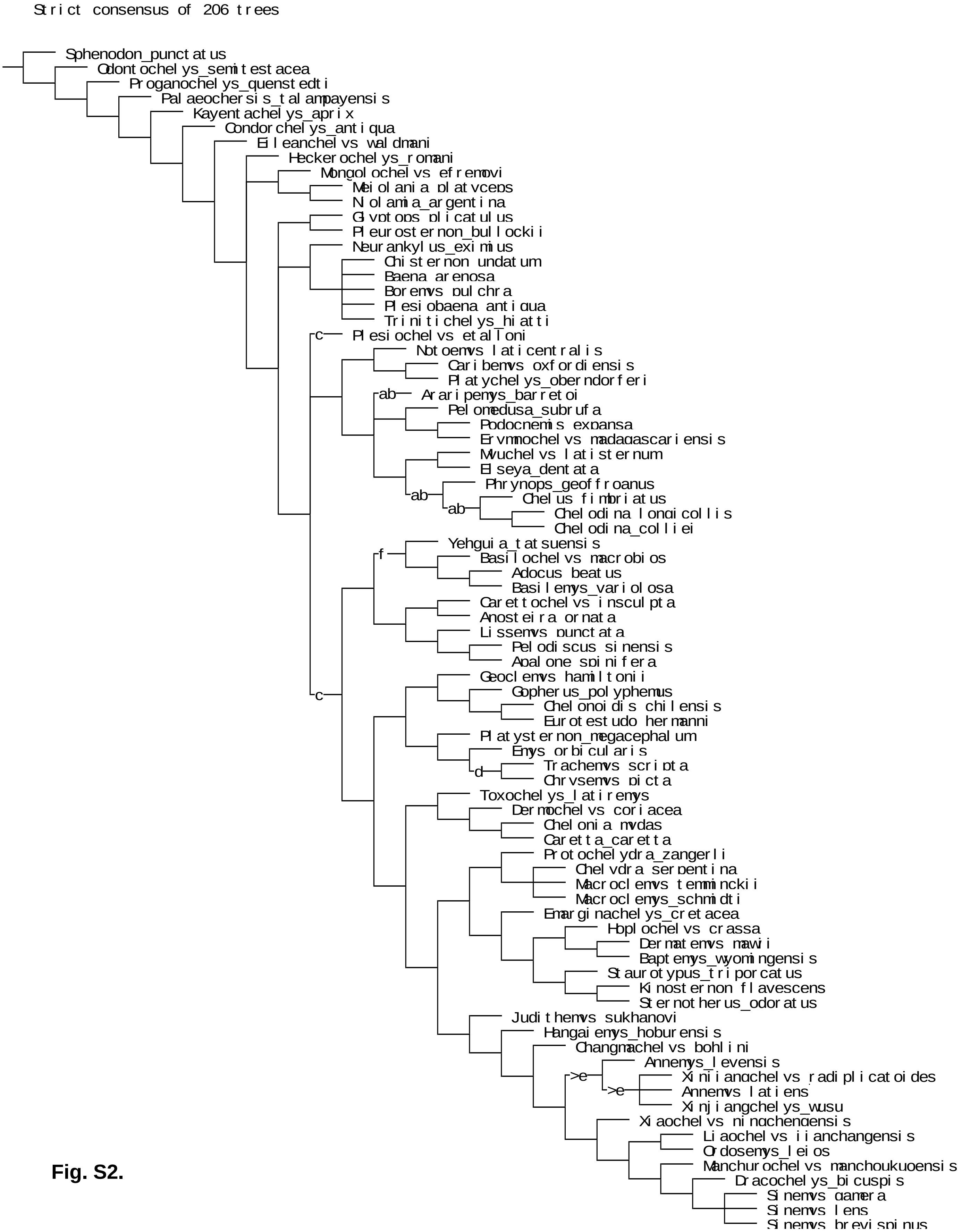
Strict consensus of 206 trees
Sphenodon_punctatus
Odontochelys_semitestacea
Proganochelys_quenstedti
Palaeochersis_talampayensis
Kayentachelys_aprix
Condorchelys_antiqua
Eileanchelys waldmani
Heckerochelys_romani
Mongolochelys efremovi
Meiolania platyceps
Niolamia_argentina
Glyptops plicatulus
Pleurosternon_bullockii
Neurankylus_eximius
Chisternon undatum
Baena arenosa
Boremys pulchra
Plesiobaena antiqua
Trinitichelys_hiatti
c
Plesiochelys etalloni
Notoemys laticentralis
Caribemys oxfordiensis
Platychelys_oberndorferi
ab
Araripemys_barretoi
Pelomedusa_subrufa
Podocnemis expansa
Erymnochelys madagascariensis
Myuchelys latisternum
Elseya_dentata
ab
Phrynops_geoffroanus
ab
Chelus fimbriatus
Chelodina longicollis
Chelodina_colliei
f
Yehguia_tatsuensis
Basilochelys macrobios
Adocus beatus
Basilemys_variolosa
Carettochelys insculpta
Anosteira ornata
Lissemys punctata
Pelodiscus sinensis
Apalone spinifera
c
Geoclemys hamiltonii
Gopherus_polyphemus
Chelonoidis chilensis
Eurotestudo hermanni
Platysternon_megacephalum
Emys orbicularis
d
Trachemys scripta
Chrysemys picta
Toxochelys_latiremys
Dermochelys coriacea
Chelonia mydas
Caretta_caretta
Protochelydra_zangerli
Chelydra serpentina
Macroclemys temminckii
Macroclemys_schmidti
Emarginachelys_cretacea
Hoplochelys crassa
Dermatemys mawii
Baptemys_wyomingensis
Staurotypus_triporcatus
Kinosternon flavescens
Sternotherus_odoratus
Judithemys sukhanovi
Hangaiemys_hoburensis
Changmachelys bohlini
>e
Annemys_levensis
>e
Xinjiangchelys radiplicatoides
Annemys latiens
Xinjiangchelys_wusu
Xiaochelys ningchengensis
Liaochelys jianchangensis
Ordosemys_leios
Manchurochelys manchoukuoensis
Dracochelys_bicuspis
Sinemys gamera
Sinemys lens
Sinemys brevispinus

Fig. S2.

Constraining *J. sukhanovi* to the stem of Kinosternoidea results in trees of 863 steps; nine steps longer than the most parsimonious trees (Fig. S3). One synapomorphy supports the clade of pan-kinosternoids and sinemydids + xinjiangchelyids + *K. hoburensis*, *C. bohlini*: presence of musk ducts.

Strict consensus of 470 trees (27 taxa excluded)

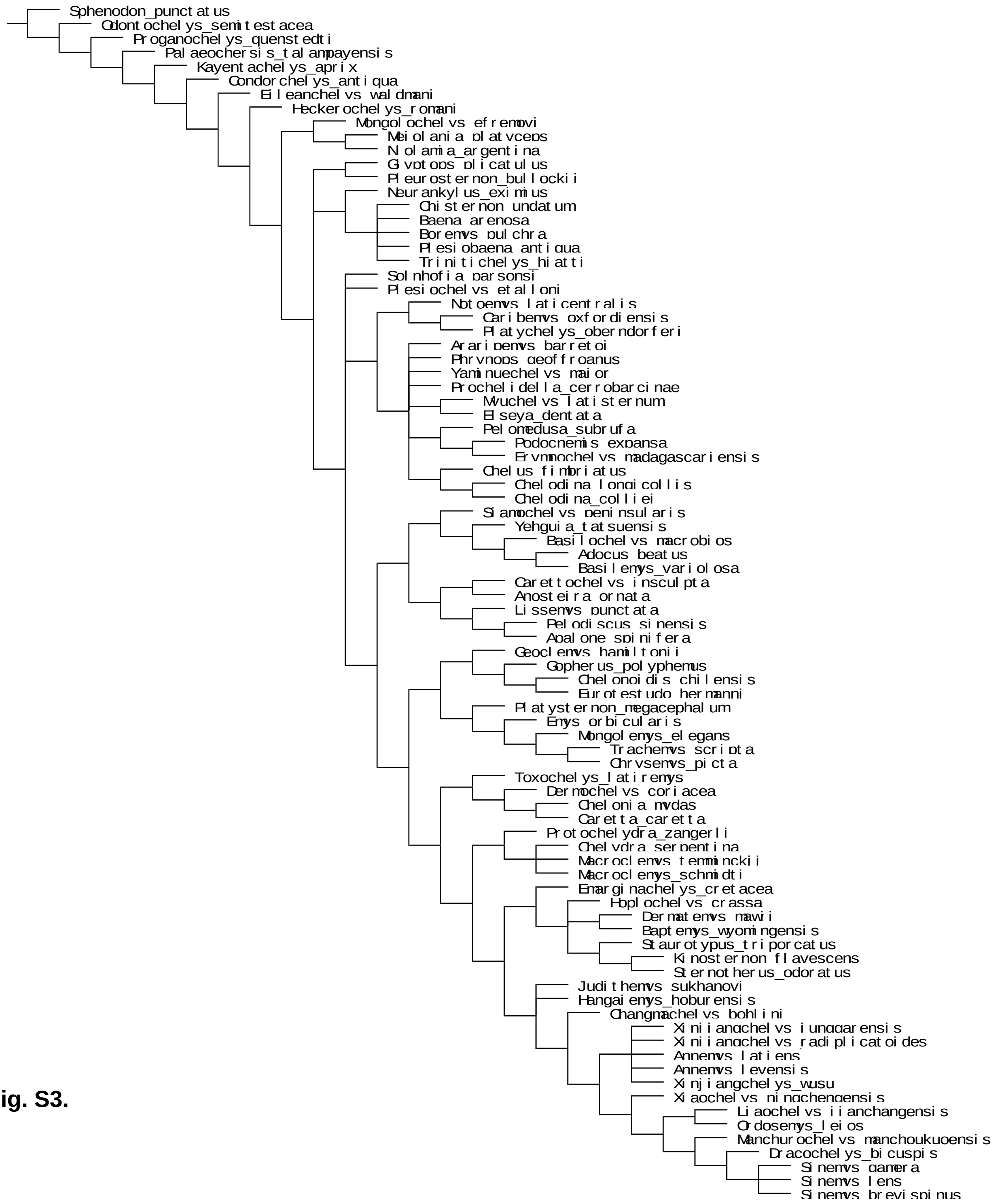

Fig. S3.

Constraining *J. sukhanovi* to the stem of Americhelydia [=(Chelonioidea (Chelydridae + Kinosternoidea)) sensu Joyce et al. 2013] results in seven extra steps (n=861) and in the placement of xinjiangchelyids, sinemydids, *Kirgizemys hoburensis*, and *Changmachelys bohlini* as stem-americhelydians (Fig. S4). The synapomorphies supporting this node are narrow and elongate epiplastra, loosely sutured entoplastron, coalescent thyroid fenestra, and a cruciform plastron. These characters are, however, polarized as primitive for crown-cryptodira in the most parsimonious trees of the unforced analysis.

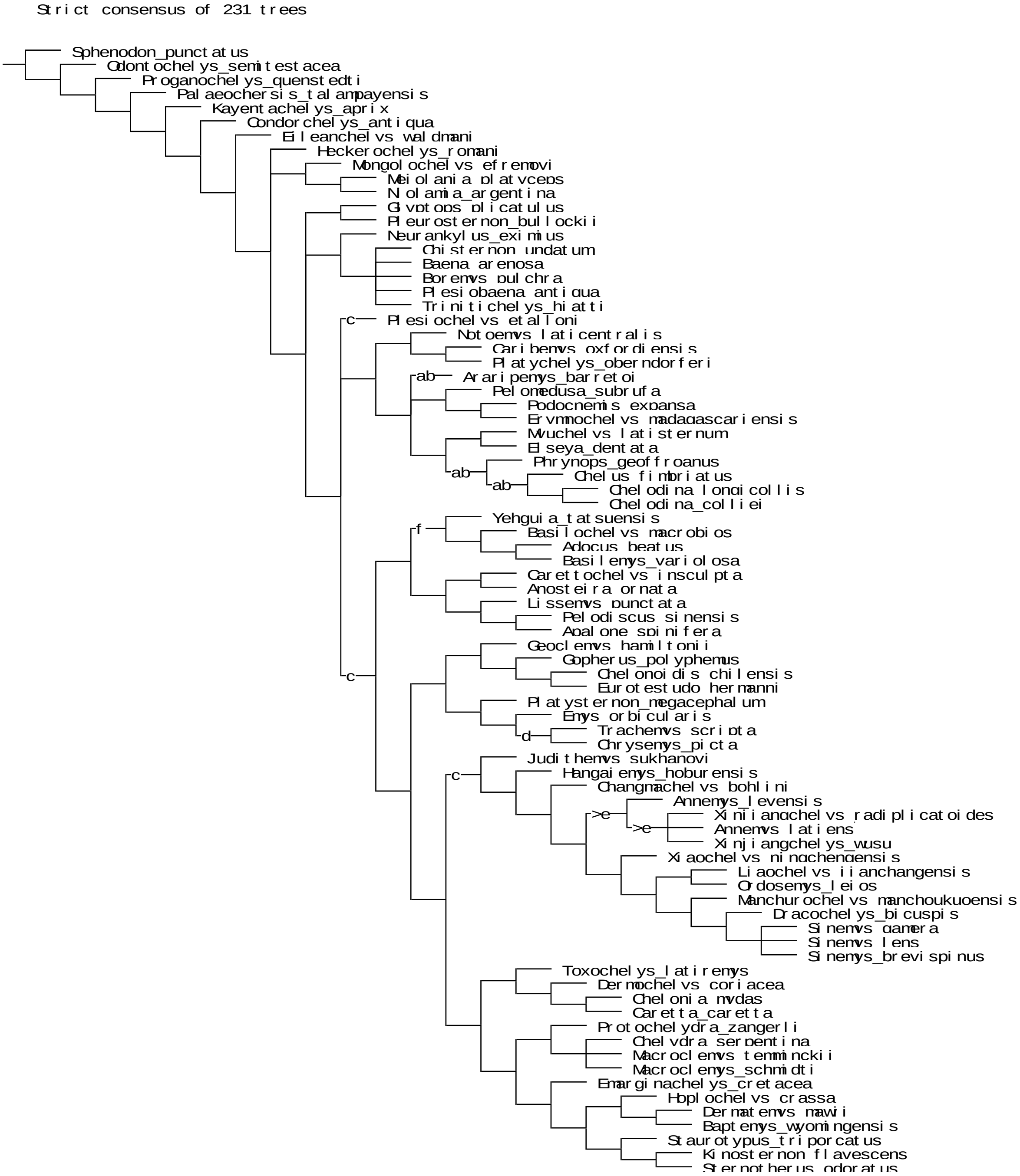

Fig. S4.

Constraining *J. sukhanovi* to the stem of Durocryptodira is resulting in nine extra steps (863 steps). *Changmachelys bohlini*, *Kirgizemys hoburensis*, Xinjiangchelyidae, and Sinemydidae are placed in the crown (to the stem of Durocryptodira) under this topological constraint Fig. S5). Synapomorphies of uniting these clades with Durocryptodira include the absence of extragular scales and a ligamentous plastron-carapace contract in some of the trees.

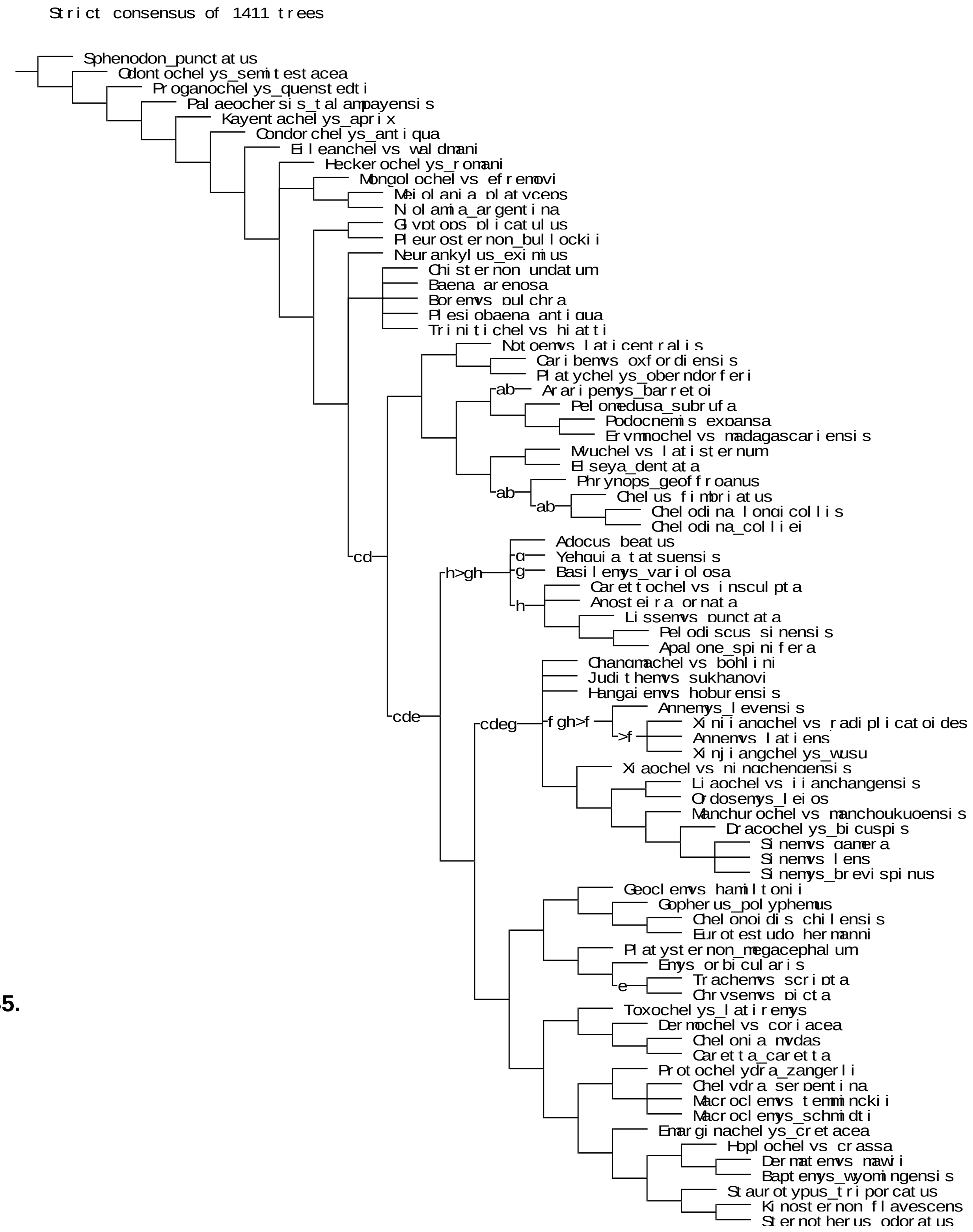

Fig. S5.

Results are similar when *J. sukhanovi* are constrained to the stem of Testudinoidea (10 extra steps, no syanpomorphies; Trionychia (7 extra steps, 3 synapomorphies: presence of peripheral gutter, junction of palatal and cerebral artery not enclosed but carotid canal ventrally from the posterior end of the skull, midline sinous plastral sulcus present), and Chelydridae (6 extra steps, 1 synapomorphy: tail as long as carapace).

Supplementary Materials

Anatomical comparisons were made based on direct observation (MR, CFZ) of all relevant Cretaceous basal eucryptodire taxa. *Dracochelys bicuspis* Gaffney and Ye, 1992 (IVPP V4075 holotype); *Kirgizemys* (=*Hangaiemys*) *hoburensis* (Sukhanov and Narmandakh, 1974) (PIN 3334-4, PIN 3334-1, PIN 3334-5, PIN 3334-16, PIN 3334-34, PIN 3334-35, PIN 3334-36, PIN 3334-37); *Judithemys sukhanovi* Parham and Hutchison, 2003 (TMP 87.2.1 holotype and material listed in Parham and Hutchison, 2003); *Liaochelys jianchangensis* Zhou, 2010a (PMOL-AR00140 holotype, PMOL-AR00160); *Manchurochelys manchoukuoensis* Endo and Shikama, 1942 (PMOL-AR00008); *Ordosemys leios* Brinkman and Peng, 1993b (IVPP V9534-1 holotype, and material listed in Brinkman and Peng 1993b); *Ordosemys liaoxiensis* Ji, 1995 (IVPP V11554; Tong et al. 2004); *Sinemys gamera* Brinkman and Peng, 1993a (IVPP V9532-1 holotype, IVPP V9532-11 and material listed in Brinkman and Peng 1993a); *Sinemys brevispinus* Tong and Brinkman, 2013 (IVPP V9538-1 holotype); *Sinemys lens* Wiman, 1930 (IVPP V8755, IVPP V9533-1), *Macrobaena mongolica* Tatarinov, 1959 (PIN 533-4, holotype).

Institutional abbreviations

IVPP, Institute of Vertebrate Paleontology and Paleoanthropology, Beijing, China; PIN, Paleontological Institute, Russian Academy of Sciences, Moscow, Russia; PMOL, Paleontological Museum of Liaoning, Shenyang Normal University, Shenyang, China; TMP, Royal Tyrrell Museum of Palaeontology, Drumheller, Canada.

Supplementary Data

Changes to the matrix of Zhou et al. (2014)

245 morphological characters and 87 taxa

See Supplementary File 1 for synapomorphies.

Nasal B: *Sinemys gamera:* 1→?

Pterygoid B: *Judithemys sukhanovi:* 2→1

Carotid circulation

Our scoring strategy for capturing the systematic variation of the characters pertaining to the carotid circulation is slightly different from that proposed by Sterli et al. (2013). In the new concept, we acknowledge the fact that many stem-cryprodires where the palatal and cerebral branches of the carotid artery are not enclosed in bone still have their carotid extending in the pterygoid from the posterior edge of the skull as in all crown-cryptodires (Rabi et al., 2013). This is fundamentally different from the morphology seen in Pan-Pleurodira where the split is covered in bone but the carotid artery is not floored from the back of the skull. Under the concept of Sterli et al. (2013), pan-pleurodires share the enclosure of the branches into bone with crown-cryptodires but that the morphologies associated with these structures are not comparable is not acknowledged. We therefore choose to give each possible conditions pertaining to the split of the cerebral and palatal branches a separate state and combine Canalis caroticum D and E into the following new character:

Carotid canal split: (0) not enclosed in bone; (1) not enclosed but carotid canal is covered ventrally from the posterior end of the skull; (2) enclosed but carotid canal is not covered

ventrally from posterior edge of skull; (3) enclosed and carotid canal covered ventrally from posterior edge of skull.

Canalis Caroticum D and **E** are omitted.

Canalis caroticum F: This character used to have three states (Sterli et al., 2013): (0) Arteria palatina enters the skull through the interpterygoid vacuity or intrapterygoid slit; (1) enters the skull through the foramen caroticum laterale between basisphenoid and pterygoid; (2) all path inside the skull. This character is reformulated to capture those taxa with an interpterygoid vacuity or slit and combines all more derived morphologies into a single state:

> **New definition:** (0) Arteria palatina enters the skull through th interpterygoid vacuity or intrapterygoid slit; (1) through foramen posterius canalis carotici palatinum or split of branches enclosed in skull.

Carapace D: *Adocus beatus:* 0→1

Carapace E: *Adocus beatus*: - →2

Nuchal B: A chelydrid state is introduced (peripheral 3 contacts cositform process). *Emarginachelys cretacea*, *Chelydra serpentina* and *Macroclemmys temminckii* are scored for this state. The character is now ordered.

Plastron A: This character is omitted because in some taxa, e.g. xinjiangchelyids and plesiochelyids a ligamentous carapace-plastron contact may look like a sutural contact in well-ossified shells (i.e. older or larger individuals) and hence difficult to score objectively for many fossil taxa known from articulated shells only. Since a revision of this matrix and a

redefinition of characters is in preparation by Rabi, Sterli and Joyce we here simply omit Plastron A.

Plastron B: *Plesiochelys etalloni:* 0→0&1 (variable based on Anquetin et al., 2014)

Plastral scutes B: *Plesiochelys etalloni:* 0→0&1 (variable or transitional morphology based on Anquetin et al., 2014)

Anal A: *Plesiochelys etalloni:* 0→0&1 (variable based on Anquetin et al., 2014)

Cervical vertebra B: *Xinjiangchelys junggarensis*: 0→1 (a ventral keel is well developed on cervical 8 based on a personal observation of IVPP V9537-1)

Cervical vertebra C: *Sinemys lens* 0→?; *Xinjiangchelys junggarensis* 0→1; *Changmachelys bohlini* ?→1

Cervical articulation A: *Xinjiangchelys junggarensis* 0→1 (based on cervical 8); *Xinjiangchelys wusu* 0→?; *Basilochelys macrobios* 0→?

Cervical articulation H: *Xinjiangchelys junggarensis* - →1

Cervical articulation L: *Xinjiangchelys junggarensis* - →0

Cervical vertebra I: State (1) is modified to: neural arch on 8th cervical modified with the postzygapophyses articular surface greatly expanded and/or pointing posteroventrally. The scoring has been modified for the following taxa: *Mesodermochelys undulatus* 0→1; *Dermochelys coriacea* 0→1; *Toxochelys latiremys*: ?→1; *Xinjiangchelys junggarensis:* 0→1; *Judithemys sukhanovi*: 0&1→1; *Dracochelys bicuspis*: 0→1; *Ordosemys leios*: 0→1; *Plesiobaena antiqua:* ?→0

Caudal C: *Mongolemys elegans:* ?→1 (based on Cadena et al., 2013)

Caudal D: Mongolemys *elegans:* ?→1 (based on Cadena et al., 2013)

Posterior plastral fontanella: *Meiolania platyceps*: 0→1; *Chelydra serpentina*: 0→1; *Macroclemmys temminckii:* 0→1; *Mongolochelys efremovi:* 0→1

Costal rib distal end: State (1) is modified to: costo-peripheral fontanelles absent, distal end of posterior dorsal ribs visible *or distal end of posterior costals narrow in dorsal view and surrounded by the peripheral.* As probably an ontogenetic variation, in some sinemydids, e.g. *Xiaochelys ningchengensis*, the distal end of the posterior ribs may not be visible due greater ossification but the corresponding costals are still narrow and the peripherals develop an unusual, proximally extending metaplastic sheet of bone to receive the costals. *Ordosemys leios*, however, exhibits none of these morphologies yet still recovered as a sinemydid.

Pterygoid I: Changed from non-applicable to 0 in all pan-pleurodires where this part of the skull is preserved.

Maxilla D: *Mongolemys elegans* 2→0 (there is only a labial ridge present).

Hyoplastron B: *Emarginachelys cretacea* ?→1; *Basilemys variolosa* ?→0

New osteological characters

Tail length: (0) tail as long as carapace; (1) tail clearly shorter than carapace.

Cruciform plastron: (0) absent; (1) present (e.g. *Chelydra serpentina*, *Kirgizemys hoburensis*, *Sternotherus odoratus*, *Chelonia mydas*, *Araripemys barretoi*).

Articulation of posterior cervical centra: (0) circular or subcircular in outline; (1) greatly flattened in outline (in coronal plane view).

Nuchal emargination (modified from Gaffney et al., 2006): (0) absent or indistinct; (1) present, excludes peripheral 1; (2) deep and involves peripheral 1 *(ordered).*

Nuchal posterior edge: (0) less than three times longer than lateral edge; (1) more than three times longer than lateral edge of nuchal.

Pterygoid extension (Gaffney and Meylan, 1988): (0) pterygoid not extending to posterior end of skull and covering prootic; (1) pterygoid extending to posterior end of skull and covering prootic.

> Comment: This character was partly included in Canalis Caroticum E which we have omitted (see explanation above).

Carotid canal entry: (0) foramen posterius canalais carotici interni is not at back of skull; (1) fpcci located at back of skull in pterygoid.

> Comment: This character is added to capture the morphology where the carotid enters at the back of the skull in the pterygoid. This character is not correlated with the character "Pterygoid extension" because paracryptodires have a posteriorly extending pterygoid but they lack a true fpcci. It is furthermore not correlated with the character "Carotid canal split".

List of characters omitted from the analysis

Pterygoid L

Comment: This character was introduced by Sterli and de la Fuente (2011) but we can not objectively reproduce their scorings and we therefore rather choose to omit it given the ongoing revision of previous global turtle matrices by Rabi, Joyce and Sterli.

Canalis caroticum D,

Canalis caroticum E

Canalis caroticum G

Comment: These characters are now redundant with the new or redefined characters introduced above.

Carapacial sutures

Comment: This character was introduced by Zhou et al. (2014) and the derived state was thought to be unique to certain sinemydids. However, our revision of other taxa revealed that it is apparently more widespread than previously recognized and therefore we see little utility in keeping it.

List of taxa included in the analysis

Sphenodon punctatus
Odontochelys semitestacea
Proganochelys quenstedti
Palaeochersis talampayensis
Condorchelys antiqua
Kayentachelys aprix
Heckerochelys romani
Eileanchelys waldmani
Mongolochelys efremovi
Meiolania platyceps
Niolamia argentina
Glyptops plicatulus

Pleurosternon bullocki
Notoemys laticentralis
Platychely oberndorferi
Caribemys oxfordensis
Prochelidella cerrobarcinae
Elysea dentata
Myuchelys latisternum
Chelodina colliei
Chelodina longicollis
Yaminuchelys maior
Phrynops geoffroanus
Chelus fimbriatus
Araripemys barretoi
Erymnochelys madagascariensis
Pelomedusa subrufa
Podocnemis expansa
Neurankylus eximius
Trinitichelys hiatti
Chisternon undatum
Plesiobaeana antiqua
Baena arenosa
Boremys pulchra
Plesiochelys etalloni
Solnhofia parsonsi
Siamochelys peninsularis
Annemys levensis
Xinjiangchelys junggarensis
Annemys latiens
Xinjiangchelys radiplicatoides
Xinjiangchelys wusu
Xiaochelys ningchengensis
Liaochelys jianchangensis
Ordosemys leios
Manchurochelys manchoukuoensis
Dracochelys bicuspis
Sinemys gamera
Sinemys lens
Sinemys brevispinus
Changmachelys bohlini
Kirgizemys hoburensis
Judithemys sukhanovi
Basilochelys macrobios

Yehguia tatsuensis
Adocus beatus
Basilemys variolosa
Carettochelys insculpta
Anosteira ornata
Lissemys punctata
Pelodiscus sinensis
Apalaone spinifera
Echmatemys wyomingensis
Geoclemys hamiltonii
Gopherus polyphemus
Chelonidis chilensis
Eurotestudo hermanni
Platysternon megacephalum
Emys orbicularis
Mongolemys elegans
Trachemys scripta
Chrysemys picta
Toxochelys latiremys
Dermochelys coriacea
Chelonia mydas
Caretta caretta
Protochelydra zangerli
Chelydra serpentina
Macrochelmys temminckii
Macroclemys schmidti
Emarginachelys cretacea
Hoplochelys crassa
Dermatemys mawii
Baptemys wyomingengsis
Staurotypus triporcatus
Kinosternon flavescens
Sternotherus odoratus

List of taxa omitted from the analysis

Simosaurus gaillardoti
Owenetta kitchingorum
Anthodon serrarius
Proterochersis robusta
Australochelys africanus

Indochelys spatulata
Ninjemys oweni
Warkalania carinaminor
Chubutemys copelloi
Kallokibotion bajazidi
Peligrochelys walshae
Patagoniaemys gasparinae
Otwayemys cunicularis
Dorsetochelys delairi
Dinochelys whitei
Portlandemys macdowelli
Thalassemys moseri
Santanachelys gaffneyi
Mesodermochelys undulatus
Chelonoidis gringorum
Stylemys nebraskensis
Xenochelys formosa
Plastomenus aff. *thomassii*
Shachemys laosiana
Chengyuchelys baenoides (IVPP V6507)
Ordosemys skull (IVPP V12092)

Fig. S1. Molecular scaffold used for parsimony analysis based on the phylogeny of Crawford et al. (2015).

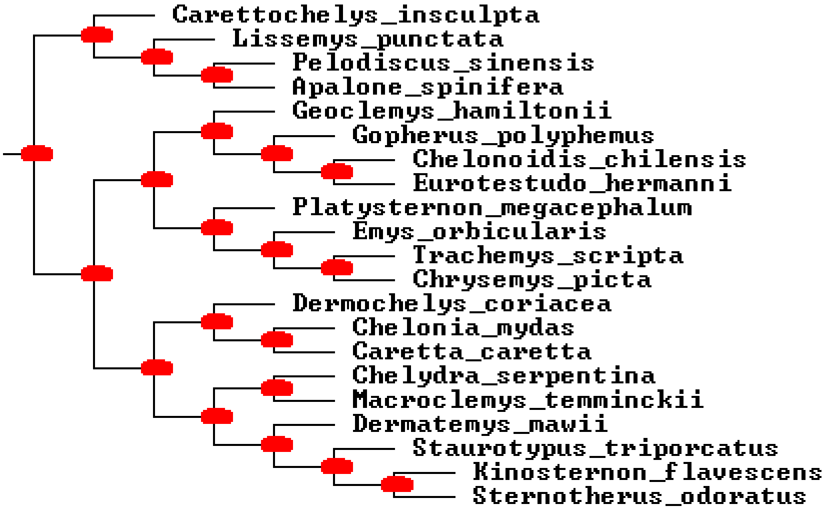

List of taxa treated as floaters in the constrained analysis

Plesiochelys etalloni
Solnhofia parsonsi
Siamochelys peninsularis
Annemys levensis
Xinjiangchelys junggarensis
Annemys latiens
Xinjiangchelys radiplicatoides
Xinjiangchelys wusu
Xiaochelys ningchengensis
Liaochelys jianchangensis
Ordosemys leios
Manchurochelys manchoukuoensis
Dracochelys bicuspis
Sinemys gamera
Sinemys lens
Sinemys brevispinus
Changmachelys bohlini
Kirgizemys hoburensis
Basilochelys macrobios
Yehguia tatsuensis
Adocus beatus
Basilemys variolosa
Echmatemys wyomingensis
Anosteira ornata
Mongolemys elegans
Toxochelys latiremys
Protochelydra zangerli
Macroclemys schmidti
Emarginachelys cretacea
Hoplochelys crassa
Baptemys wyomingengsis

Supplementary References

Anquetin, J., Püntener, C., & Billon-Bruyat, J. P. A taxonomic review of the Late Jurassic eucryptodiran turtles from the Jura Mountains (Switzerland and France). *PeerJ* **2**, e369 (2014).

Brinkman, D. B. & Peng, J.-H. New material of *Sinemys* (Testudines, Sinemydidae) from the Early Cretaceous of China. *Canadian Journal of Earth Sciences.* **30**, 2139-2152 (1993a).

Brinkman, D. B. & Peng, J.-H. *Ordosemys leios*, n. gen., n. sp., a new turtle from the Early Cretaceous of the Ordos Basin, Inner Mongolia. *Canadian Journal of Earth Sciences.* **30**, 2128-2138 (1993b).

Cadena, E. A., Ksepka, D. T., & Norell, M. A. New material of *Mongolemys elegans* Khosatzky and Mlynarski, 1971 (Testudines: Lindholmemydidae), from the Late Cretaceous of Mongolia with comments on bone histology and phylogeny. *American Museum Novitates* 3766, 1-28 (2013).

Crawford, N. G., Parham, J. F., Sellas, A. B., Faircloth, B. C., Glenn, T. C., Papenfuss, T. J., & Simison, W. B. A phylogenomic analysis of turtles. *Molecular phylogenetics and evolution* 83, 250-257 (2015).

Endo, R. & Shikama, R. Mesozoic reptilian fauna in the Jehol Mountainland, Manchoukuo. *Buletin of the Central National Museum of Manchoukuo* **3**, 1-20 (1942).

Gaffney, E. S., & Meylan, P. A. A phylogeny of turtles. *In:* The Phylogeny and Classification of the Tetrapods, Volume 1: Amphibians, Reptiles, Birds (M. J. Benton, Ed) pp. 157-219, Systematics Association Special Volume No. 35A, Clarendon Press, Oxford (1988).

Gaffney, E. S. & Ye, X.-K. *Dracochelys*, a new cryptodiran turtle from the Early Cretaceous of China. *American Museum Novitates* **3048**, 1-13 (1992).

Gaffney, E. S., Tong, H., & Meylan, P. A. Evolution of the side-necked turtles: the families Bothremydidae, Euraxemydidae, and Araripemydidae. *Bulletin of the American Museum of Natural History,* 1-698 (2006).

Ji, S.-A. Reptiles. *In:* Fauna and Stratigraphy of Jurassic-Cretaceous in Beijing and the Adjacent Areas (Ren D, Lu L-W, Guo Z-G, Ji S-A, Eds) pp. 140-146, Seismic Press, Beijing (1995).

Parham, J. F. & Hutchison, J. H. A new eucryptodiran turtle from the Late Cretaceous of North America (Dinosaur Provincial Park, Alberta, Canada). *Journal of Vertebrate Paleontology* **23**, 783-798 (2003).

Pritchard, P. C. H. Encyclopedia of turtles. T.F.H. Publications, Hong Kong (1979).

Rabi, M., Zhou, C. F., Wings, O., Ge, S., & Joyce, W. G. A new xinjiangchelyid turtle from the Middle Jurassic of Xinjiang, China and the evolution of the basipterygoid process in Mesozoic turtles. *BMC evolutionary Biology*, **13**(1), 203 (2013).

Sterli, J., & De La Fuente, M. S. A new turtle from the La Colonia Formation (Campanian–Maastrichtian), Patagonia, Argentina, with remarks on the evolution of the vertebral column in turtles. *Palaeontology*, 54(1), 63-78.(2011).

Sterli, J., Pol, D., & Laurin, M. Incorporating phylogenetic uncertainty on phylogeny-based palaeontological dating and the timing of turtle diversification. *Cladistics* 29, 233-246 (2013).

Sukhanov, V. B. & Narmandakh, P. [A new Early Cretaceous turtle from the continental deposits of the Northern Gobi (in Russian)] Mesozoic and Cenozoic Faunas and Biostratigraphy of Mongolia. *Transactions of the. Joint Soviet Mongolian Paleontological Expedition* **1**, 192-220 (1974).

Tatarinov, L. P. A new turtle of the family Baenidae from the Lower Eocene of Mongolia. *Paleontologicheskiy Zhurnal* **1**, 100-113 (1959). [in Russian]

Tong, H. & Brinkman, D. A new species of *Sinemys* (Testudines: Cryptodira: Sinemydidae) from the Early Cretaceous of Inner Mongolia, China. *Palaeobio. Palaeoenv.* **93**, 355-366 (2013).

Tong, H., Ji, S.-A. & Ji, Q. *Ordosemys* (Testudines: Cryptodira) from the Yixian Formation of Liaoning Province, northeastern China: new specimens and systematic revision. *Am. Mus. Novit.* **3438**, 1-20 (2004).

Wiman, C. Fossile Schildkroten aus China. *Palaeontologia Sinica, Series C*, **6**, 5-53 (1930).

Zhou, C.-F. A new eucryptodiran turtle from the Early Cretaceous Jiufotang Formation of western Liaoning, China. *Zootaxa* **2676**, 45-56 (2010a).

Zhou, C.-F., Rabi, M., & Joyce, W. G. A new specimen of Manchurochelys manchoukuoensis from the Early Cretaceous Jehol Biota of Chifeng, Inner Mongolia, China and the phylogeny of Cretaceous basal eucryptodiran turtles. *BMC Evolutionary Biology* **14**, 77 (2014).

Synapomorphy Tree

Synapomorphies common to 1469 trees

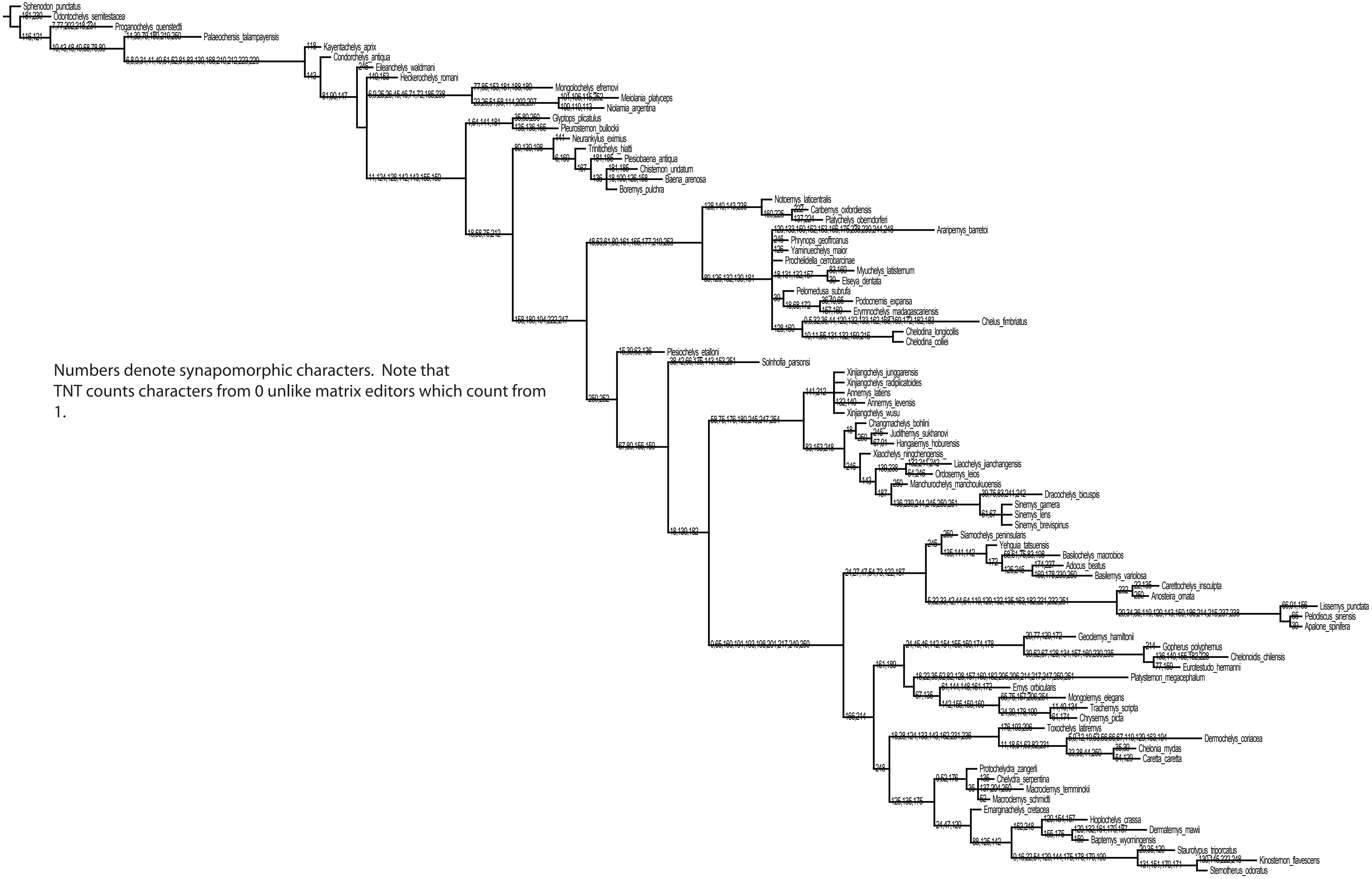

Numbers denote synapomorphic characters. Note that TNT counts characters from 0 unlike matrix editors which count from 1.
